# Regular physical activity prevents development of chronic muscle pain through modulation of supraspinal opioid and serotonergic mechanisms

**DOI:** 10.1097/PR9.0000000000000618

**Published:** 2017-08-21

**Authors:** Renan G. Brito, Lynn A. Rasmussen, Kathleen A. Sluka

**Affiliations:** Department of Physical Therapy and Rehabilitation Science, Pain Research Program, University of Iowa, Iowa City, IA, USA

**Keywords:** Pain, Analgesia, Exercise, Physical activity, Serotonin, Opioid, Periaqueductal gray, Rostral ventromedial medulla, Serotonin transporter, SERT, Supraspinal

## Abstract

**Introduction:**

It is generally believed that exercise produces its effects by activating central opioid receptors; there are little data that support this claim. The periaqueductal gray (PAG) and rostral ventromedial medulla (RVM) are key nuclei in opioid-induced analgesia, and opioids interact with serotonin to produce analgesia.

**Objectives:**

The purpose was to examine central inhibitory mechanisms involved in analgesia produced by wheel running.

**Methods:**

C57/Black6 mice were given access to running wheels in their home cages before induction of chronic muscle hyperalgesia and compared with those without running wheels. Systemic, intra-PAG, and intra-RVM naloxone tested the role of central opioid receptors in the antinociceptive effects of wheel running in animals with muscle insult. Immunohistochemistry for the serotonin transporter (SERT) in the spinal cord and RVM, and pharmacological blockade of SERT, tested whether the serotonin system was modulated by muscle insult and wheel running.

**Results:**

Wheel running prevented the development of muscle hyperalgesia. Systemic naloxone, intra-PAG naloxone, and intra-RVM naloxone reversed the antinociceptive effect of wheel running in animals that had received muscle insult. Induction of chronic muscle hyperalgesia increased SERT in the RVM, and blockade of SERT reversed the hyperalgesia in sedentary animals. Wheel running reduced SERT expression in animals with muscle insult. The serotonin transporter in the superficial dorsal horn of the spinal cord was unchanged after muscle insult, but increased after wheel running.

**Conclusion:**

These data support the hypothesis that wheel running produced analgesia through central inhibitory mechanisms involving opioidergic and serotonergic systems.

## Abstract 2

The current study shows that blockade of opioid receptors systemically in the periaqueductal gray and the rostral ventromedial medulla prevents analgesia by 8 weeks of wheel running in a chronic muscle pain model. We further show increases in serotonin transporter expression and reversal of hyperalgesia with a selective reuptake inhibitor in the rostral ventromedial medulla in the chronic muscle pain model, and exercise normalizes serotonin transporter expression.

## 1. Introduction

People who are physically active are less likely to develop chronic musculoskeletal pain.^48,49,111^ Similarly, previous studies by us and others show that prior physical activity prevents development of hyperalgesia in animal models of muscle and neuropathic pain.^6,29,77,94^ Thus, these data suggest that the underlying mechanisms by which physical activity prevents development of pain are key factors for the transition from acute to chronic pain.

Although it is generally believed that exercise produces its effects by activating central opioid receptors, there are little data that support this claim. In healthy human subjects, a single exercise task increases beta-endorphin and produces analgesia that is blocked by naloxone.^37,82^ In uninjured animals, regular exercise produces tolerance at mu-opioid receptors, and analgesia is blocked by opioid receptor antagonists.^37,44,60,62,97,98^ In animals with neuropathic pain, treadmill exercise started after injury, increases endogenous opioids in the periaqueductal gray (PAG) (β-endorphin) and the rostral ventromedial medulla (RVM) (met-enkephalin), and both systemic and intracerebroventricular naloxone reverses the antinociception.^101^ In animals with chronic muscle pain, treadmill running starting after development of hyperalgesia produces antinociception that is reversed by systemic naloxone.^5^ Furthermore, in animals with nerve injury, the serotonin transporter (SERT) is increased, and serotonin is decreased in the RVM, both of which are normalized by treadmill running starting after injury,^6^ and systemic serotonergic depletion prevents this exercise-induced analgesia.^6,63^These studies suggest that opioids and serotonin play a role in analgesia produced by exercise in human subject and animals with and without pain, and suggest that central pain inhibitory pathways underlie this analgesia.

The RVM mediates hyperalgesia after tissue insult, as well as opioid-induced analgesia, and PAG-mediated opioid analgesia.^9,16,25,31,36,71,74,102–106,108^ For example, systemic morphine analgesia is reduced by lesions or opioid receptor blockade in the PAG,^20,22^ and analgesia produced by activation of the PAG is prevented by opioid receptor blockade or lesions of the RVM.^4,25,27,46,79,100,110^ Further opioid receptors and opioid peptides are located in the RVM, and activation of opioid receptors in the RVM produces analgesia.^1,24,36,39,43,72,73^ Furthermore, serotonergic RVM neurons receive input from endogenous opioid peptides and project to the spinal cord to produce analgesia, or hyperalgesia, depending on the receptor activated.^63^ In the RVM, microinjection of 5-HT produces analgesia, and mice lacking central 5-HT neurons show greater inflammatory hyperalgesia.^56,57,113^ Furthermore, systemic serotonergic depletion, or blockade of serotonin receptors in the RVM, prevents analgesia from morphine injected systemically or into the PAG.^11,45,81^ These data show a critical role for the PAG and RVM in endogenous inhibition, that this, analgesia involves opioids and serotonin.

The purpose of the current study was to examine the central opioid sites involved in the prevention of chronic muscle pain by regular physical activity, if SERT expression in the RVM and spinal cord is modulated by regular physical activity, and if SERT mediates the hyperalgesia in animals with chronic muscle pain.

## 2. Material and methods

### 2.1. Sedentary vs physically active mice

All experiments were approved by the Animal Care and Use Committee at the University of Iowa. Male (n = 42) and female (n = 30) C57BL/6 mice were used for these experiments. Sedentary and naive mice were housed in their home cage with food and water. Physically active mice had free access to running wheels in their home cages for 8 weeks, and activity, in km/d, was recorded. Mice were housed individually. Running wheels were removed from home cages after 8 weeks, before induction of the chronic muscle pain model. We previously showed that 8 weeks of running wheel activity prevents development of hyperalgesia in this model that lasts for 8 days after stopping the running wheel activity.^93^

### 2.2. Noninflammatory chronic muscle pain

The model of chronic muscle pain was induced by 2 injections of unbuffered pH 4.0 saline (20 μL) into the left gastrocnemius muscle 5 days apart while the mouse was anesthetized with 4% isoflurane, as previously published.^93^ This insult produces enhanced mechanical sensitivity of the paw and muscle bilaterally without tissue damage.^18,93^

### 2.3. Behavior testing

Mice were acclimated for 2×/day for 2 days prior to behaviour testing procedures. This included acclimation to a gardener's glove used to immobilize the animals, acclimation to the wire mesh in small Lucite cubicles, and acclimation to the grip force test. All experiments were performed with the experimenter blinded to the group.

Muscle withdrawal thresholds were tested by applying a pair of calibrated forceps to the gastrocnemius muscle as previously described and validated.^89,107^ Mice were placed in a gardener's glove, and one hindlimb was extended. A pair of forceps was applied perpendicularly to the middle of the gastrocnemius muscle belly until the animal withdrew the hindlimb. Tests were performed bilaterally and 3 trials averaged.

The response frequency to mechanical stimulation of the paw was tested as previously described.^18^ The number of withdrawals to a von Frey filament (2.44, 0.4 mN) applied to the paw 5 times was assessed, followed by a 5-minute waiting period. This was repeated 10 times, averaged, and presented as percent response.

Grip strength of the hindpaw and forepaw was tested as previously described.^10^ Mice were pulled by the tail until a good grip was felt to read grip force for either the hindpaw or the forepaw. An average of 5 trials was recorded.

### 2.4. Implantation of guide cannula

Intracerebral guide cannulae were placed in the vlPAG and RVM 2 days before the first intramuscular injection of 4.0 as previously described.^21,92^ Mice were anesthetized with ketamine/xylazine and positioned in a stereotaxic head holder. The skull was exposed, and a small hole drilled for placement of guide cannulae. For the vlPAG, cannula were placed −4.1 mm from bregma, 0.4 mm from midline, and −2.6 mm from surface; for the RVM, cannula were placed −5.6 mm from bregma, 0.0 mm from midline, and −5.7 mm from surface. Cannulae were secured to the skull with 2 screws and dental cement. Mice were allowed to recover 3 to 7 days before testing. To examine the placement of the cannula, 0.2 μL methylene blue dye was injected through the cannula at the end of the experiment. After euthanasia, the brain was removed and postfixed in 10% formalin. The day before cutting, brains were transferred to 30% sucrose. Forty micrometer sections were cryosectioned through the injection site and examined under light microscopy for the placement of the cannula. Sites were verified by 2 individuals and recorded in a spreadsheet. In additional mice, guide cannula were misplaced outside the RVM, into the gigantocellularis nucleus (n = 8), and outside the vlPAG into the dorsolateral PAG (n = 2), lateral PAG (n = 1), or deep mesencephalic nucleus (n = 1).

### 2.5. Drugs

Naloxone hydrochloride and naloxone methiodide (Sigma-Aldrich, St. Louis, MO; 10 mg/kg, i.p.) were dissolved in 0.9% sterile saline for systemic delivery, and dosing was based on previously published data in mice.^67,68,70,109^ For microinjection, naloxone hydrochloride was dissolved in saline at a concentration of 10 nmol/0.2 μL; dosing was based on previously published data in mice.^12,14^ Fluoxetine doses (Sigma-Aldrich) were extrapolated from previous reports and tested in preliminary studies.^13,58^ For fluoxetine, 2 nmol/0.2 μL was dissolved in 0.9% sterile saline and 20 nmol/0.2 μL in 10% 2-hydroxypropyl-B-cyclodextrin (HBC) in sterile saline. Vehicles for all experiments were 0.9% sterile saline, except for the 20 nmol/0.2 μL, which used 10% HBC in sterile saline.

### 2.6. Immunohistochemistry

Animals were deeply anesthetized with 150 mg/kg sodium pentobarbital and transcardially perfused with heparinized saline followed by 4% paraformaldehyde. The brainstem and lumbar spinal cord was removed, stored in 30% sucrose overnight, and frozen at −20°C. Sections were cut on a cryostat at 20 μm on to slides. All sections were stained using an antibody to SERT (rabbit Anti-5HT Transporter; 1:1000) using standard immunofluorescent techniques as previously described.^6^ Preliminary data show specificity of the antibody to SERT because removal of the primary antibody and SERT−/− mice showed no immunoreactivity.^54^ On day 1, sections were blocked in 3% normal goat serum, avidin, and biotin and incubated in the primary antibody overnight at room temperature. On day 2, sections were rinsed, blocked in 3% normal goat serum, and incubated in biotinylated immunoglobulin G (IgG) for 1 hour at room temperature (1:1,000; Invitrogen, Eugene, OR). These sections were then reacted with streptavidin conjugated to Alexa Fluor 568 (Goat Anti-rabbit IgG; 1:1000) for 1 hour at room temperature. Sections were coverslipped with Vectashield (Vector Labs, Burlingame, CA) and stored until analysis. Brain and spinal cord sections for each group were stained simultaneously to avoid variability between stains across days.

Images of stained sections were taken in the Central Microscopy Facility at the University of Iowa on an Olympus BX-51 light microscope equipped with a SPOT camera (RT Slider; Diagnostic Instruments, Sterling Heights, MI). Rostral ventromedial medulla sections were identified at Bregma −6.00 mm from the Mouse Brain Atlas.^69^ All sections were taken under the same condition and stored for later analysis. Using Image J software, optical density was analyzed as previously described.^38^ Each picture was converted to 8-bit gray scale, calibrated independently using the “uncalibrated OD” function, and density values were analyzed per area. Ten sections of the RVM and superficial dorsal horn were quantified.

### 2.7. Experimental design

Experiment 1 tested whether a regular physical activity prevented development of chronic muscle hyperalgesia using the following groups: (1) sedentary with muscle insult (n = 3 M, 3 F), (2) wheel running with muscle insult (n = 4 M, 4 F), and (3) naive (n = 3 M, 3 F). Mice were tested before wheel running, and before and 24 hours after muscle insult for muscle withdrawal thresholds, paw response frequency, and grip strength. Naive mice did not receive muscle insult and did not have access to running wheels. Running wheels were stopped at the time of the first intramuscular injection.

Experiment 2 tested the role of peripheral and central opioid systems on prevention of hyperalgesia by wheel running using the following groups: (1) vehicle (n = 3 M, 2 F), (2) naloxone hydrochloride (10 mg/kg, i.p.; n = 3 M, 2 F), and (3) naloxone methiodide (10 mg/kg, i.p.; n = 2 M, 3 F). Muscle withdrawal thresholds were assessed before and 24 hours after muscle insult; and 30 and 120 minutes after injection. Running wheels were stopped at the time of the first intramuscular injection. Naloxone or vehicle was administered 24 hours after the second intramuscular injection.

Experiment 3 tested whether the PAG and RVM, central opioid sites involved in analgesia, were involved in the prevention of hyperalgesia by wheel running. For PAG, active mice with muscle insult were divided in 2 groups: (1) vehicle (saline, n = 3 M, 3 F) and (2) naloxone hydrochloride (10 nmol/0.2 μL, i.c.; n = 4 M, 3 F). For RVM, active animals with the muscle insult were divided into 2 groups: (1) vehicle (n = 3 M, 2 F) and (2) naloxone hydrochloride (10 nmol/0.2 μL, i.c.; n = 3 M, 3 F). Muscle withdrawal thresholds were assessed before and 24 hours after muscle insult, and 30 and 120 minutes after microinjection. Running wheels were stopped at the time of cannulae placement. Two days after the placement of cannulae, the first intramuscular injection of acidic saline was given. Naloxone or vehicle was administered 24 hours after the second intramuscular injection.

Experiment 4 tested the involvement of the serotonin system in the chronic muscle pain model and modulation by wheel running. Using animals from experiment 1, SERT, expression in the RVM, and superficial dorsal horn using immunohistochemistry in sedentary, physically active, and naive mice was assessed.

Experiment 5 tested whether pharmacological blockade of SERT in the RVM, in animals with muscle insult, mimicked the behavioural effects of exercise. Sedentary mice were implanted with guide cannula in RVM, induced with chronic muscle pain model, and randomly divided as follows: (1) Saline (n = 5 M, 1 F), (2) Saline with 10% HBC (n = 3 M, 2 F), (3) Fluoxetine (2 nmol/0.2 μL) (n = 4 M, 2 F), and (4) Fluoxetine (20 nmol/0.2 μL) (n = 3 M, 3 F). Muscle withdrawal thresholds were tested before and 24 hours after muscle insult; and 30 and 120 minutes after microinjection.

### 2.8. Statistical analysis

A repeated-measures analysis of variance was performed to test for changes in withdrawal thresholds of the muscle, the response frequency to mechanical stimulation of the paw, and grip force to examine for differences between groups and across time. This was followed by post hoc testing with a Tukey's test for differences between groups. Experiment 1 had 3 groups (sedentary, wheel running, and naive) and 3 time periods (baseline, before insult, and after insult). Experiment 2 had 3 groups (vehicle, naloxone hydrochloride, and naloxone methiodide) and 4 time periods (before and after muscle insult, 30 minutes and 1 hour after drug). Experiment 3 had 2 groups per site (vehicle and naloxone hydrochloride) and 4 time periods (before and after muscle insult, 30 minutes and 1 hour after drug). Experiment 5 had 5 groups (saline, saline with 10% HBC, fluoxetine 2 nmol, fluoxetine 20 nmol, missed sites) and 4 time periods before and after muscle insult, 30 minutes and 1 hour after drug). We report within-subjects effects, within-subjects contrasts, and between-subjects effects for group for each experiment in Table 1. Post hoc analysis examined for individual differences between groups with Tukey's test. For immunohistochemistry in experiment 4, a 1-way analysis of variance tested for differences between groups for the changes in SERT expression which had 3 groups (sedentary, wheel running, and naive) and 1 time period followed by post hoc analysis for group differences with Tukey's test. All data are mean ± SEM, and *P* ≤ 0.05 is considered significant.

**Table 1:**
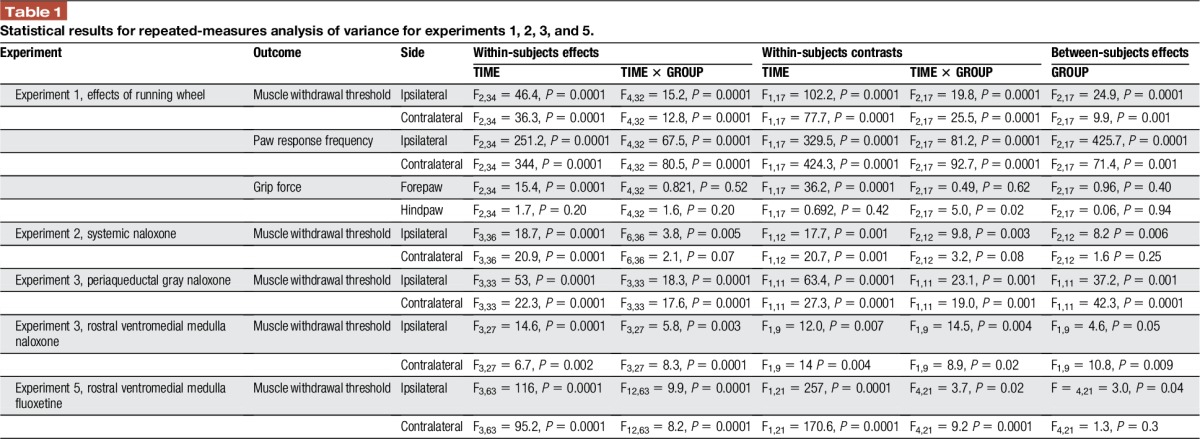
Statistical results for repeated-measures analysis of variance for experiments 1, 2, 3, and 5.

## 3. Results

### 3.1. Physical activity prevents development of chronic muscle hyperalgesia

**Figure 1.**
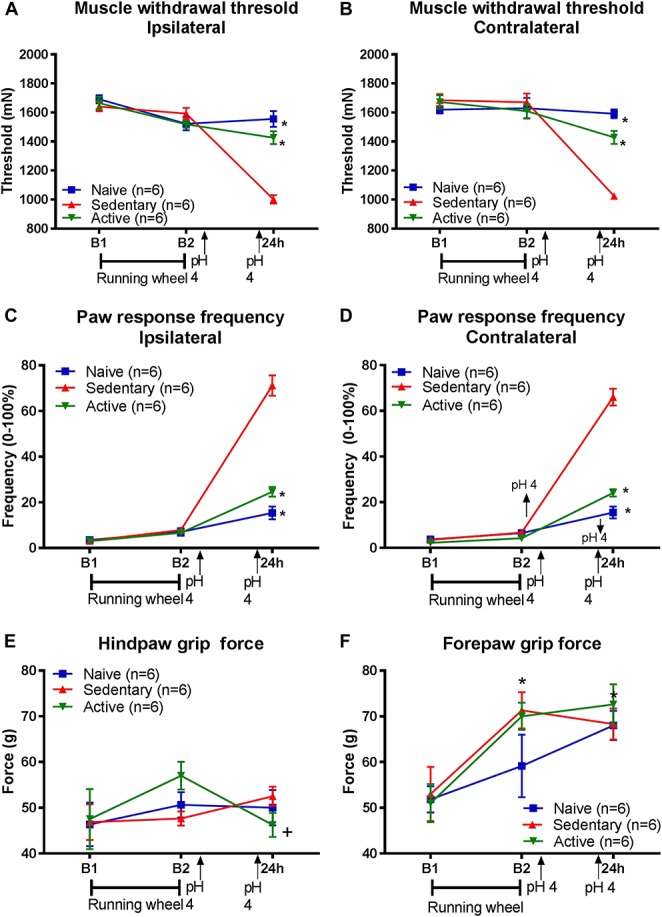
Long-term running wheel activity prevents development of hyperalgesia produced by repeated acid injections. Graphs show the muscle withdrawal threshold (A and B), paw response frequency (C and D), and grip force (E and F) before (B1) and after 8 weeks of running wheel (B2), and 24 hours after the second injection of acidic saline for naive (blue), sedentary (red), and running wheel (active, green) groups. The withdrawal threshold and paw response frequency were significantly lower in sedentary animals after the second acid injection when compared with the naive or physically active groups bilaterally (*P < 0.05). The hind paw grip force was significantly greater in the active group compared with the naive group for the forepaw and significantly less after the second acidic saline injection for the active group (*, *P* < 0.05) compared with the time after running wheel (+, *P* < 0.05). Data are the mean ± SEM.

On average, mice ran 2.46 ± 0.87 km/d over the 8-week time period with a range from 0.86 km/d to 9.48 km/d. There was a significant overall difference in muscle withdrawal thresholds for time, an interaction between time and group, and a significant difference between groups (Table 1). Specifically, 24 hours after induction of the chronic muscle pain model, the sedentary mice showed significantly lower muscle withdrawal thresholds when compared with the physically active mice (ipsilateral: *P* = 0.0001, contralateral: *P* = 0.02) and the naive mice (ipsilateral: *P* = 0.0001; contralateral: *P* = 0.0001) (Fig. 1A, B).

For response frequency of the paw, there was a significant overall difference for time, an interaction between time and group, and a significant difference between groups (Table 1). Specifically, 24 hours after induction of the chronic muscle pain model, the sedentary mice showed a greater frequency of response to repeated mechanical stimulation of the paw bilaterally when compared with the physically active mice (ipsilateral, contralateral: *P* = 0.0001) and naive mice (ipsilateral, contralateral: *P* = 0.0001) (Fig. 1C, D).

The forepaw showed a significant difference in grip force across time, but no interaction between time and group, and no group differences (Table 1). In the active group, there were significant increases in grip force after 8 weeks of wheel running (*P* = 0.02, paired to-test) that were maintained 24 hours after induction of the chronic muscle pain model (*P* = 0.0001). The hindpaw grip force showed a significant interaction between time and group, but no difference for time (Table 1). In the active group, hindpaws showed significant decreases in force after induction of the chronic pain model (*P* = 0.001) when compared with the value 8 weeks after wheel running (Fig. 1E, F).

### 3.2. Central opioids mediate analgesia produced by wheel running

**Figure 2.**
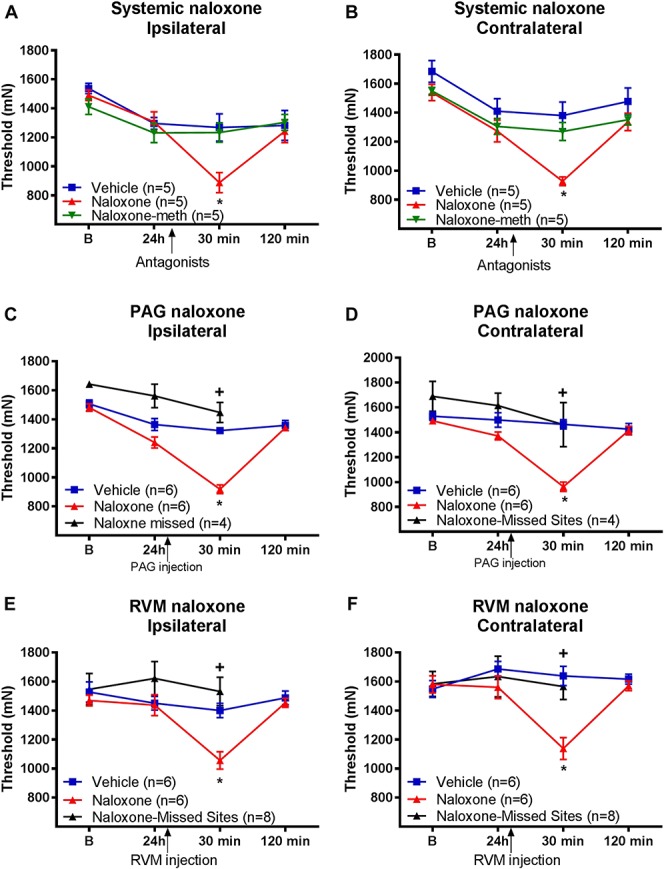
Central opioid receptors in periaqueductal gray (PAG) and rostral ventromedial medulla (RVM) mediate the analgesia produced by wheel running. Muscle withdrawal thresholds for animals treated with naloxone systemically (A and B), into the PAG (C and D) and into the RVM (E and F) for both the ipsilateral and contralateral sides. All animals were given running wheels for 8 weeks before induction of the chronic muscle pain model. Thirty minutes after injection of naloxone systemically, or into the PAG or RVM, there was a significant decrease in the withdrawal thresholds of the muscle (*, *P* < 0.05) when compared with animals that received vehicle. Missed injection sites at the level of the PAG or RVM show significantly greater withdrawal thresholds compared with those injected with naloxone into the PAG or RVM (+, *P* < 0.05). Data are the mean ± SEM.

As opioids have been reported to mediate the analgesic effects of exercise,^5,101^ we tested if systemic naloxone reversed the analgesic effects of wheel running 24 hours after muscle insult. We compared this to the peripherally restricted opioid antagonist, naloxone methiodide, to examine contributions of peripheral opioid receptors. There was a significant overall difference for time, an interaction between time and group, and a significant difference between groups (Table 1). The naloxone-treated group was significantly lower than the saline-treated group (ipsilateral: *P* = 0.01; contralateral *P* = 0.001). Naloxone methiodide had no effect on the analgesia and was not different from the saline-treated group (ipsilateral: *P* = 0.94; contralateral: *P* = 0.50) (Fig. 2A, B).

Because systemic naloxone, but not naloxone methiodide, reversed the analgesic effects of regular physical activity, we tested whether central sites were involved in the antinociception. Because the PAG also uses opioids to produce analgesia,^25^ we tested whether blockade of opioid receptors in the PAG reversed the antinociceptive effects of wheel running. Microinjection of naloxone hydrochloride into the vlPAG 24 hours after muscle insult reversed the antinociceptive effects of wheel running. There was a significant overall difference for time, an interaction between time and group, and a significant difference between groups (Table 1). Specifically, the withdrawal thresholds of the muscle were significantly lower in the physically active mice that received naloxone in the PAG when compared with the saline group (ipsilateral, contralateral: *P* = 0.0001) 30 minutes after injection (Fig. 2C, D). This effect was temporary, and withdrawal thresholds were not significantly different from the saline group 2 hours after induction of the hyperalgesia (ipsilateral: *P* = 0.7; contralateral: *P* = 0.52) (Fig. 2C, D). Furthermore, 4 animals had cannulae misplaced outside the vlPAG into the dlPAG, lPAG and deep mesencephalic nucleus. In these animals, the withdrawal threshold remained unchanged after microinjection of naloxone hydrochloride, and was significantly greater than in those injected with naloxone into the vlPAG (Fig. 2C, D) (*P* < 0.05).

Because the RVM plays a significant role in analgesia, receives projections from the PAG, and uses opioid receptors to produce analgesia,^25^ we tested whether opioid receptors in the RVM were involved in the analgesic effect of wheel running after muscle insult. Microinjection of naloxone into the RVM 24 hours after induction of the chronic muscle pain model reversed the antinociceptive effects of regular physical activity. There was a significant overall difference for time, an interaction between time and group, and a significant difference between groups (Table 1). Specifically, after microinjection of naloxone into the RVM, muscle withdrawal thresholds were significantly lower than those that received saline (ipsilateral: *P* = 0.002; contralateral: *P* = 0.001) (Fig. 2E, F). The effect was temporary, and antinociception returned 2 hours after naloxone injection (vs vehicle; ipsilateral: *P* = 0.54; contralateral: *P* = 0.36) (Fig. 2E, F). In 8 mice, naloxone hydrochloride was injected into the gigantocellularis nucleus (Gi). In these animals, there was no change in withdrawal thresholds after microinjection, and these were significantly different than withdrawal thresholds from animals injected with naloxone into the RVM (Fig. 2E, F) (*P* < 0.05).

### 3.3. Physical activity modulates serotonin transporter expression

**Figure 3.**
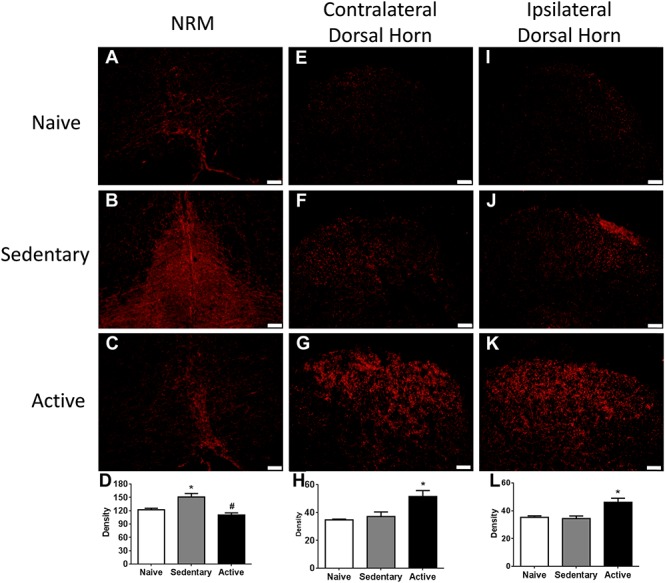
Wheel running modulates expression of serotonin transporter (SERT) in the RVM and spinal cord. Representative photomicrographs of the nucleus raphe magnus (NRM, A-C) and spinal cord dorsal horn (ipsilateral: E-G; contralateral: I-K) from naive, sedentary, and active animals immunohistochemically stained for SERT. In the NRM, there was an increase in staining density in the sedentary animals (B and D) when compared with the naive (A and D) that was reduced in the exercised group (C and D). Quantitation of the density of staining showed a significant increase in staining density in the sedentary group in the NRM (*, *P* < 0.05) that was significantly reduced in the physically active animals (#, *P* < 0.05). For the dorsal horn, there was minimal immunohistochemical staining for SERT in the superficial dorsal horn in naive and sedentary mice. In the physically active animals, there was an increase in staining density in the superficial dorsal horn bilaterally. Quantification showed a significant increase in staining density in the active group when compared with sedentary or naive mice (*, *P* < 0.05). Data are the mean ± SEM.

Because there is an interaction between the serotonergic and opioidergic systems in the RVM,^11,45^ we tested whether there were changes in SERT in the RVM. Figures 3A and D shows SERT staining density in the nucleus raphe magnus (NRM) 24 hours after muscle insult in sedentary animals compared with those that had performed wheel running and naive controls. After muscle insult, there were significant differences in the density of SERT immunoreactivity in the NRM (F_2,18_ = 13.1, *P* = 0.0001), with sedentary mice showing an increase in SERT density 24 hours after muscle insult when compared with naive controls (*P* = 0.009). Furthermore, SERT density in the physically active mice was significantly lower than that in sedentary mice 24 hours after muscle insult (*P* = 0.0001), but not different from naive mice (*P* = 0.35).

Because the RVM projects to the spinal cord, and serotonin plays a role in pain inhibition in the spinal cord,^3,20,25^ we tested whether there were differences in SERT in the spinal cord. Figures 3E and L shows the SERT staining density in the spinal cord 24 hours after muscle insult in sedentary animals and those that performed 8 weeks of wheel running compared with naive controls. There was a significant difference in the density of immunoreactivity for SERT bilaterally in the spinal cord dorsal horn (ipsilateral: F_2,18_ = 9.9, *P* = 0.002; contralateral: F_2,18_ = 7.4.1, *P* = 0.005). There was no significant difference between sedentary mice in SERT density in the spinal cord 24 hours after muscle insult when compared with naive controls (ipsilateral: *P* = 0.97; contralateral: *P* = 0.88). However, in animals that performed wheel running and received muscle insult, SERT density was significantly greater bilaterally than naive (ipsilateral: *P* = 0.006; contralateral: *P* = 0.007) or sedentary mice 24 hours after muscle insult (ipsilateral: *P* = 0.003; contralateral: *P* = 0.02).

### 3.4. Blockade of serotonin transporter reverses hyperalgesia

**Figure 4.**
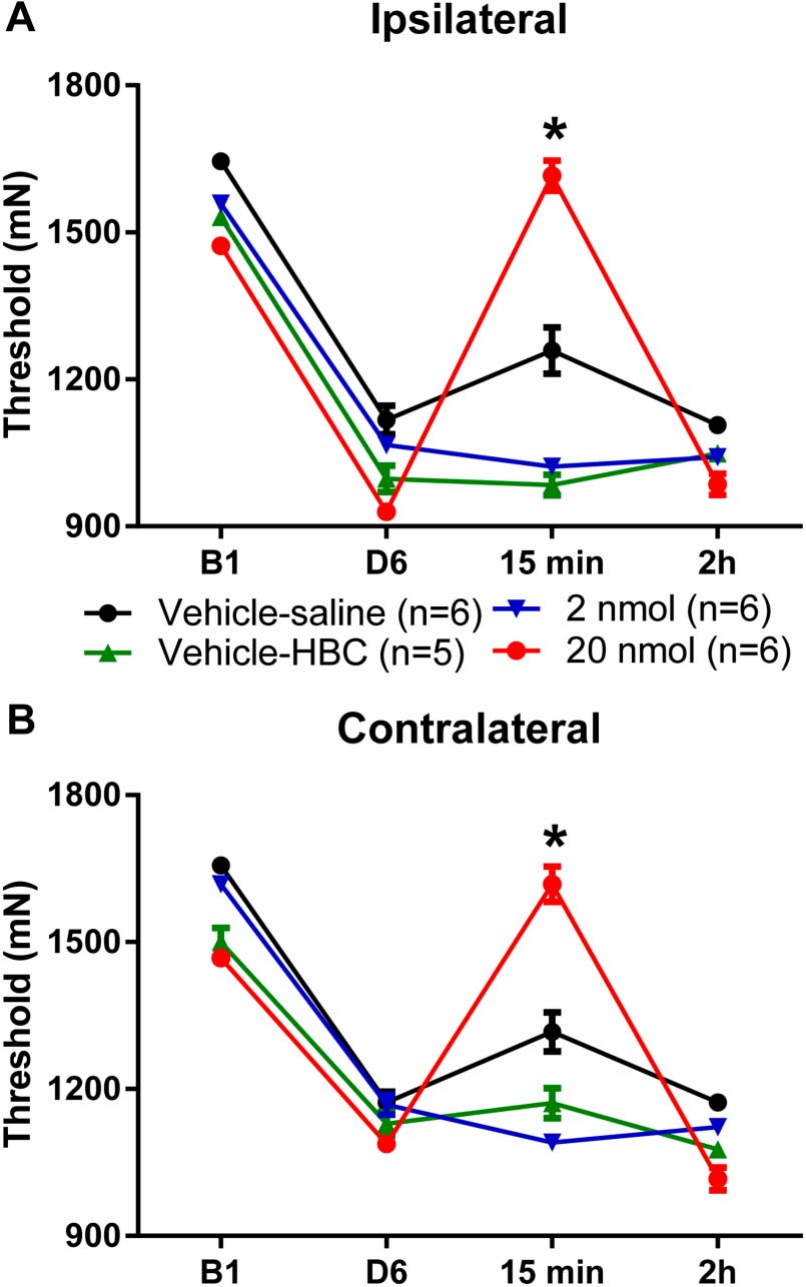
Blockade of serotonin transporter reverses hyperalgesia produced by repeated acid injections. Graphs represent muscle withdrawal thresholds before and 24 hours after the second acid injection in sedentary animals. The decrease in withdrawal threshold of the paw induced by repeated acid injection was reversed 15 minutes after microinjection of 20 nmol/0.2 μL of fluoxetine (*, *P* < 0.05) for both the ipsilateral (A) and contralateral (B) hindlimbs. By 2 hours after injection, the hyperalgesia returned to preinjection thresholds. Data are the mean ± SEM.

Because there were increases in SERT in the RVM after muscle insult and regular physical activity decreases these SERT increases, we tested whether blockade of SERT in the RVM of sedentary animals with muscle insult mimicked the effects of wheel running. Fluoxetine was microinjected into the RVM 24 hours after induction of the model. For these experiments, there was a significant difference in withdrawal thresholds of the muscle, a significant overall difference of time, an interaction between time and group, and a significant difference between groups (Table 1). Muscle withdrawal thresholds from the group that received 20 nmol fluoxetine were significantly greater than those from the group that received saline (ipsilateral: *P* = 0.017; contralateral: *P* = 0.001), 2 nmol fluoxetine (ipsilateral: *P* = 0.0001), and HBC-vehicle (ipsilateral: *P* = 0.0001; contralateral: *P* = 0.004) 15 minutes after injection (Fig. 4). Fluoxetine effects were temporary, and by 2 hours, there were no significant differences between groups. In 3 animals that received 2 nmol dose of fluoxetine, the cannula were misplaced and withdrawal thresholds were decreased bilaterally (ipsilateral: 1172 + 43 mN; contralateral: 1246 + 15.6 mN).

## 4. Discussion

The current study showed that blockade of opioid receptors in the RVM and PAG reversed the antinociception produced by long-term wheel running before muscle insult. These data are in agreement with prior studies in healthy human subjects showing an increase in systemic β-endorphin, and blockade of analgesia with systemic naloxone, after high-intensity exercise.^7,15,33,82^ In animals without tissue injury, systemic naloxone reduces analgesia produced by strength training, treadmill exercise, and long-term running wheel activity.^26,53,86,101^ Furthermore, long-term wheel running also decreased analgesia to exogenously applied mu-opioid agonists,^44,60,61^ suggesting that opioid tolerance had developed. Thus, prior studies in human subjects and animal without tissue injury, clearly show a role for opioids in analgesia using not only high-intensity exercise, but also lower-intensity tasks like wheel running.

More recent studies evaluated effects of opioid antagonism on exercise-induced analgesia in animal models of pain. In these studies, systemic naloxone reversed analgesia produced by moderate- to low-intensity exercise started after development of hyperalgesia in animals with neuropathic pain, chronic muscle pain, and in those that exercised before induction of acetic acid–induced pain.^5,59,62,83,101^ In parallel, animals with neuropathic pain that perform moderate intensity treadmill exercise, after nerve injury, show increases β-endorphin in the PAG and met-enkephalin in the RVM, and supraspinal blockade of opioids (intracerebroventricularly) prevents the exercise-induced analgesia.^101^ On the other hand, blockade of peripheral opioid receptors has no effect on exercise-induced analgesia in animals with neuropathic pain^101^; the current study shows similar results.

The current study also showed that naloxone in either the PAG or the RVM reversed the antinociception produced by regular physical activity, and this effect was temporary. This would suggest that exercise does not prevent central sensitization that occurs after repeated acid injections, but that endogenous opioids produced by exercise decrease nociceptive sensitivity. While the PAG and RVM are involved in pain inhibition, they are also involved in pain facilitation, particularly in chronic musculoskeletal pain models.^21,102^ It is therefore also possible that the increases in endogenous opioids in PAG and RVM are part of a parallel and independent pathway masking the ongoing central sensitization. Thus, increases in central opioids, within the PAG and RVM, mediate the analgesia by regular exercise through a modifiable mechanism.

Serotonin is a major neurotransmitter found in endogenous inhibitory pathways including the RVM and spinal cord and plays a significant role in analgesia. Classical studies show that increasing 5-HT in the NRM, by direct microinjection of 5-HT or a reuptake inhibitor, produces analgesia.^40,56,57^ By contrast, blockade of 5-HT2B receptors in the NRM prevents fear-induced analgesia,^19^ and morphine-induced analgesia.^45,56^ These data suggest that, in the RVM, increases in serotonin are analgesic, decreases in serotonin are hyperalgesic, and serotonergic analgesia involves the opioidergic system. Serotonin transporters control extracellular levels of 5-HT, thereby regulating serotonergic transmission.^51^ The current study showed that increases in SERT in the RVM were observed 24 hours after induction of the chronic muscle pain model, suggesting increased removal of released serotonin. These studies are in agreement with our prior study showing decreased 5-HT, and increases in SERT, in the NRM in animals with neuropathic pain.^6^ We extended these prior studies by showing that pharmacological blockade of SERT in the RVM reverses hyperalgesia in this model, providing a physiological role for SERT in the hyperalgesia that develops in chronic muscle pain. Depletion of serotonin with p-chlorophenylalanine prevents exercise-induced analgesia in healthy animals and in those that exercised after the development of with neuropathic pain,^6,62^ supporting the proposal that an increase in serotonin mediates the analgesia by exercise. Indeed, both our current and prior studies^6^ show decreases in SERT expression in the RVM in exercised animals, whether exercising before or after injury, suggesting regulation of SERT expression by regular exercise. It is unknown, whether the decreases in SERT are specific to animals with hyperalgesia, or whether these changes are observed in uninjured animals that exercise. Serotonin transporter surface expression is dynamically regulated by intracellular signalling pathways. Protein kinase C phosphorylation increases SERT internalization, and p-38 phosphorylation increases surface expression.^50,51,76,78^ Thus, SERT expression in chronic pain conditions may be dynamically modulated by regular physical activity through the release of endogenous opioids.

Serotonin fibers in the spinal cord originate in the RVM,^8^ and stimulation of the PAG or RVM increases 5-HT spinally.^34,99^ The current study shows no change in SERT immunoreactivity in the spinal cord after the induction of the chronic muscle pain model, suggesting similar levels of serotonin in the chronic muscle pain model. However, physically active mice show increased SERT in the superficial dorsal horn, suggesting that there is less serotonin available in the spinal cord in exercised animals. The literature on spinal serotonin is varied, with some studies showing a role for serotonin in inhibition and others in facilitation of nociception.^28,30,32,41,84^ Whether serotonin is inhibitory or excitatory in the spinal cord depends on the serotonin receptor activated, the animal model used, or the analgesic modality. Illustrating the variability, intrathecal injection of methysergide, a 5-HT2 antagonist, reduces hyperalgesia in nerve injured mice^80^ and prevents analgesia by electrical stimulation of the RVM, morphine injected into the RVM or PAG, and transcutaneous electrical nerve stimulation.^2,35,41,75^ Furthermore, spinal blockade of 5-HT1 receptors reverses hyperalgesia produced by inflammation^111^ and prevents analgesia produced by electrical stimulation of the RVM or by joint manipulation.^23,88^ Thus, future studies will need to investigate whether the increases in SERT in the spinal cord play a functional role in exercise-induced analgesia.

The chronic muscle pain model used in the current study produces bilateral hyperalgesia of the muscle and paw, visceral hyperalgesia, and anxiety-like and depressive-like behaviors.^55,65,84,93^ Once it develops, hyperalgesia is maintained by central mechanisms^93^ that include sensitization of spinal dorsal horn neurons manifested by bilateral expansion of receptive fields,^95^ and activation of central facilitatory pathways in the PAG, RVM, and spinal cord.^17,38,87,102^ The chronic muscle pain model has a similar pharmacological profile to fibromyalgia, a centrally mediated clinical pain condition, with analgesic effects using centrally acting drugs, eg, reuptake inhibitors, but not peripherally acting drugs, eg, nonsteroidal anti-inflammatory drugs.^47,64,66,96^ Thus, this animal model produces widespread pain similar to that observed in fibromyalgia that involves sensitization of central nervous system pathways.^91^

In conclusion, long-term voluntary wheel running produces analgesia through activation of endogenous inhibitory mechanisms that include opioids and serotonin, confirming several long-standing theories for how exercise produces analgesia. We propose that exercise resets the nervous system, so that subsequently painful stimuli normally perceived as painful in sedentary individuals, are not perceived as painful in active individuals. We suggest that there is a balance between inhibition and excitation in the central nervous system that favours excitation in sedentary individuals resulting in development of pain, and favours inhibition in physically active individuals preventing development of chronic pain. Exercise has the capacity to increase endogenous inhibition, which is reduced in individuals with chronic pain,^42,52,90^ and thus may be a therapy that can alter central processing of nociception.

## Disclosures

The authors have no conflict of interest to declare.

This work was supported by the National Institutes of Health grant AR061371.

## References

[1] Arvidsson U Riedl M Chakrabarti S Lee JH Nakano AH Dado RJ Loh HH Law PY Wessendorf MW Elde R Distribution and targeting of a mu-opioid receptor (MOR1) in brain and spinal cord. J Neurosci 1995;15:3328–41. 7751913 10.1523/JNEUROSCI.15-05-03328.1995 PMC6578209

[2] Barbaro NM Hammond DL Fields HL Effects of intrathecally administered methysergide and yohimbine on microstimulation-produced antinociception in the rat. Brain Res 1985;343:223–9. 2996695 10.1016/0006-8993(85)90738-3

[3] Basbaum AI Descending control of pain transmission: possible serotonergic-enkephalinergic interactions. Adv Exp Med Biol 1981;133:177–89. 6172014 10.1007/978-1-4684-3860-4_9

[4] Basbaum AI Clanton CH Fields HL Opiate and stimulus produced analgesia: functional anatomy of a medullospinal pathway. Proc Natl Acad Sci U S A 1976;73:4685–8. 1070018 10.1073/pnas.73.12.4685 PMC431597

[5] Bement MK Sluka KA Low-intensity exercise reverses chronic muscle pain in the rat in a naloxone-dependent manner. Arch Phys Med Rehabil 2005;86:1736–40. 16181935 10.1016/j.apmr.2005.03.029

[6] Bobinski F Ferreira TA Cordova MM Dombrowski PA da CC Santo CC Poli A Pires RG Martins-Silva C Sluka KA Santos AR Role of brainstem serotonin in analgesia produced by low-intensity exercise on neuropathic pain after sciatic nerve injury in mice. PAIN 2015;156:2595–606. 26447701 10.1097/j.pain.0000000000000372 PMC4666012

[7] Bortz WM Angwin P Mefford IN Boarder MR Noyce N Barchas JD Catecholamines, dopamine, and endorphin levels during extreme exercise. N Engl J Med 1981;305:466–7. 7254293

[8] Bowker RM Westlund KN Sullivan MC Wilber JF Coulter JD Descending serotonergic, peptidergic and cholinergic pathways from the raphe nuclei: a multiple transmitter complex. Brain Res 1983;288:33–48. 6198030 10.1016/0006-8993(83)90079-3

[9] Burgess SE Gardell LR Ossipov MH Malan TP Vanderah TW Lai J Porreca F Time-dependent descending facilitation from the rostral ventromedial medulla maintains, but does not initiate, neuropathic pain. J Neurosci 2002;22:5129–36. 12077208 10.1523/JNEUROSCI.22-12-05129.2002 PMC6757729

[10] Burnes LA Kolker SJ Danielson JF Walder RY Sluka KA Enhanced muscle fatigue occurs in male but not female ASIC3-/- mice. Am J Physiol Regul Integr Comp Physiol 2008;294:R1347–55. 18305024 10.1152/ajpregu.00687.2007 PMC2746663

[11] Carruba MO Nisoli E Garosi V Sacerdote P Panerai AE da PM Catecholamine and serotonin depletion from rat spinal cord: effects on morphine and footshock induced analgesia. Pharmacol Res 1992;25:187–94. 1635896 10.1016/1043-6618(92)91387-v

[12] Cavun S Resch GE Evec AD Rapacon-Baker MM Millington WR Blockade of delta opioid receptors in the ventrolateral periaqueductal gray region inhibits the fall in arterial pressure evoked by hemorrhage. J Pharmacol Exp Ther 2001;297:612–19. 11303050

[13] Cervantes-Duran C Rocha-Gonzalez HI Granados-Soto V Peripheral and spinal 5-HT receptors participate in the pronociceptive and antinociceptive effects of fluoxetine in rats. Neuroscience 2013;252:396–409. 23994595 10.1016/j.neuroscience.2013.08.022

[14] Chu X Xu N Li P Mao L Wang JQ Inhibition of cardiovascular activity following microinjection of novel opioid-like neuropeptide nociceptin (orphanin FQ) into the rat rostral ventrolateral medulla. Brain Res 1999;829:134–42. 10350539 10.1016/s0006-8993(99)01357-8

[15] Colt EW Wardlaw SL Frantz AG The effect of running on plasma beta-endorphin. Life Sci 1981;28:1637–40. 7242250 10.1016/0024-3205(81)90319-2

[16] Coutinho SV Urban MO Gebhart GF Role of glutamate receptors and nitric oxide in the rostral ventromedial medulla in visceral hyperalgesia. PAIN 1998;78:59–69. 9822212 10.1016/S0304-3959(98)00137-7

[17] da Silva LFS DeSantana JM Sluka KA Activation of NMDA receptors in the brainstem, rostral ventromedial medulla, and nucleus reticularis gigantocellularis mediates mechanical hyperalgesia produced by repeated intramuscular injections of acidic saline in rats. PAIN 2010;11:378–87. 10.1016/j.jpain.2009.08.006 PMC2933661 19853525

[18] da Silva LFS Walder RY Davidson BL Wilson SP Sluka KA Changes in expression of NMDA-NR1 receptor subunits in the rostral ventromedial medulla modulates pain behaviors. PAIN 2010;151:155–61. 20688433 10.1016/j.pain.2010.06.037 PMC2943935

[19] de OR de Oliveira RC Falconi-Sobrinho LL da Silva SR Jr Coimbra NC 5-Hydroxytryptamine2A/2C receptors of nucleus raphe magnus and gigantocellularis/paragigantocellularis pars alpha reticular nuclei modulate the unconditioned fear-induced antinociception evoked by electrical stimulation of deep layers of the superior colliculus and dorsal periaqueductal grey matter. Behav Brain Res 2017;316:294–304. 27616344 10.1016/j.bbr.2016.09.016

[20] Deakin JF Dostrovsky JO Involvement of the periaqueductal grey matter and spinal 5-hydroxytryptaminergic pathways in morphine analgesia: effects of lesions and 5-hydroxytryptamine depletion. Br J Pharmacol 1978;63:159–65. 206302 10.1111/j.1476-5381.1978.tb07785.x PMC1668289

[21] DeSantana JM da Silva LF De Resende MA Sluka KA Transcutaneous electrical nerve stimulation at both high and low frequencies activates ventrolateral periaqueductal grey to decrease mechanical hyperalgesia in arthritic rats. Neuroscience 2009;163:1233–41. 19576962 10.1016/j.neuroscience.2009.06.056 PMC3955259

[22] Dostrovsky JO Deakin JF Periaqueductal grey lesions reduce morphine analgesia in the rat. Neurosci Lett 1977;4:99–103. 19604928 10.1016/0304-3940(77)90151-3

[23] El-Yassir N Fleetwood-Walker SM A 5-HT_1_ receptor mediates the antinociceptive effect of nucleus raphe magnus stimulation in the rat. Brain Res 1990;523:92–9. 2207694 10.1016/0006-8993(90)91639-x

[24] Fang FG Haws CM Drasner K Williamson A Fields HL Opioid peptides (DAGO-enkephalin, dynorphin A(1-13), BAM 22P) microinjected into the rat brainstem: comparison of their antinociceptive effect and their effect on neuronal firing in the rostral ventromedial medulla. Brain Res 1989;501:116–28. 2572306 10.1016/0006-8993(89)91033-0

[25] Fields HL Basbaum AI Heinricher MM Central nervous system mechanisms of pain modulation. In: McMahon SB Koltzenburg M, editors. Textbook of pain. Philadelphia: Elsevier, 2006 pp. 125–42.

[26] Galdino GS Duarte ID Perez AC Participation of endogenous opioids in the antinociception induced by resistance exercise in rats. Braz J Med Biol Res 2010;43:906–9. 20802976 10.1590/s0100-879x2010007500086

[27] Gebhart GF Sandkuhler J Thalhammer JG Zimmermann M Inhibition of spinal nociceptive information by stimulation in midbrain of the cat is blocked by lidocaine microinjected in nucleus raphe magnus and medullary reticular formation. J Neurophysiol 1983;50:1446–59. 6663337 10.1152/jn.1983.50.6.1446

[28] Goodchild CS Guo Z Freeman J Gent JP 5-HT spinal antinociception involves mu opioid receptors: cross tolerance and antagonist studies. Br J Anaesth 1997;78:563–9. 9175973 10.1093/bja/78.5.563

[29] Grace PM Fabisiak TJ Green-Fulgham SM Anderson ND Strand KA Kwilasz AJ Galer EL Walker FR Greenwood BN Maier SF Fleshner M Watkins LR Prior voluntary wheel running attenuates neuropathic pain. PAIN 2016;157:2012–23. 27355182 10.1097/j.pain.0000000000000607 PMC4988888

[30] Gravitt K Marson L Effect of the destruction of cells containing the serotonin reuptake transporter on urethrogenital reflexes. J Sex Med 2007;4:322–30. 17367427 10.1111/j.1743-6109.2007.00436.x

[31] Guan Y Guo W Robbins MT Dubner R Ren K Changes in AMPA receptor phosphorylation in the rostral ventromedial medulla after inflammatory hyperalgesia in rats. Neurosci Lett 2004;366:201–5. 15276247 10.1016/j.neulet.2004.05.051

[32] Guo W Miyoshi K Dubner R Gu M Li M Liu J Yang J Zou S Ren K Noguchi K Wei F Spinal 5-HT3 receptors mediate descending facilitation and contribute to behavioral hypersensitivity via a reciprocal neuron-glial signaling cascade. Mol Pain 2014;10:35. 24913307 10.1186/1744-8069-10-35 PMC4067691

[33] Haier RJ Quaid K Mills JC Naloxone alters pain perception after jogging. Psychiatry Res 1981;5:231–2. 6945616 10.1016/0165-1781(81)90052-4

[34] Hammond DL Tyce GM Yaksh TL Efflux of 5-hydroxytryptamine and noradrenaline into spinal cord superfusates during stimulation of the rat medulla. J Physiol (London) 1985;359:151–62. 2582112 10.1113/jphysiol.1985.sp015579 PMC1193369

[35] Hammond DL Yaksh TL Antagonism of stimulation-produced antinociception by intrathecal administration of methysergide or phentolamine. Brain Res 1984;298:329–37. 6326954 10.1016/0006-8993(84)91432-x

[36] Heinricher MM Schouten JC Jobst EE Activation of brainstem N-methyl-D-aspartate receptors is required for the analgesic actions of morphine given systemically. PAIN 2001;92:129–38. 11323134 10.1016/s0304-3959(00)00480-2

[37] Hoeger Bement MK Sluka KA Exercise-induced hypoalgesia: an Evidence-based review. In: Sluka KA, editor. Pain mechanisms and management for the physical therapist. Philadelphia: Wolters Kluwer, 2016 pp. 177–202.

[38] Hoeger-Bement MK Sluka KA Phosphorylation of CREB and mechanical hyperalgesia is reversed by blockade of the cAMP pathway in a time-dependent manner after repeated intramuscular acid injections. J Neurosci 2003;23:5437–45. 12843242 10.1523/JNEUROSCI.23-13-05437.2003 PMC6741249

[39] Hurley RW Hammond DL The analgesic effects of supraspinal mu and delta opioid receptor agonists are potentiated during persistent inflammation. J Neurosci 2000;20:1249–59. 10648729 10.1523/JNEUROSCI.20-03-01249.2000 PMC6774182

[40] Inase M Nakahama H Otsuki T Fang JZ Analgesic effects of serotonin microinjection into nucleus raphe magnus and nucleus raphe dorsalis evaluated by the monosodium urate (MSU) tonic pain model in the rat. Brain Res 1987;426:205–11. 3690323 10.1016/0006-8993(87)90874-2

[41] Jensen TS Yaksh TL Examination of spinal monoamine receptors through which brainstem opiate-sensitive systems act in the rat. Brain Res 1986;363:114–27. 3004638 10.1016/0006-8993(86)90663-3

[42] Julien N Goffaux P Arsenault P Marchand S Widespread pain in fibromyalgia is related to a deficit of endogenous pain inhibition. PAIN 2005;114:295–302. 15733656 10.1016/j.pain.2004.12.032

[43] Kalyuzhny AE Wessendorf MW Relationship of mu- and delta-opioid receptors to GABAergic neurons in the central nervous system, including antinociceptive brainstem circuits. J Comp Neurol 1998;392:528–47. 9514515

[44] Kanarek RB Gerstein AV Wildman RP Mathes WF D'Anci KE Chronic running-wheel activity decreases sensitivity to morphine-induced analgesia in male and female rats. Pharmacol Biochem Behav 1998;61:19–27. 9715803 10.1016/s0091-3057(98)00059-8

[45] Kiefel JM Cooper ML Bodnar RJ Serotonin receptor subtype antagonists in the medial ventral medulla inhibit mesencephalic opiate analgesia. Brain Res 1992;597:331–8. 1473004 10.1016/0006-8993(92)91490-6

[46] Kiefel JM Rossi GC Bodnar RJ Medullary mu and delta opioid receptors modulate mesencephalic morphine analgesia in rats. Brain Res 1993;624:151–61. 8252387 10.1016/0006-8993(93)90073-v

[47] Kim SH Song J Mun H Park KU Effect of the combined use of tramadol and milnacipran on pain threshold in an animal model of fibromyalgia. Korean J Intern Med 2009;24:139–42. 19543493 10.3904/kjim.2009.24.2.139 PMC2698623

[48] Landmark T Romundstad P Borchgrevink PC Kaasa S Dale O Associations between recreational exercise and chronic pain in the general population: evidence from the HUNT 3 study. PAIN 2011;152:2241–7. 21601986 10.1016/j.pain.2011.04.029

[49] Landmark T Romundstad PR Borchgrevink PC Kaasa S Dale O Longitudinal associations between exercise and pain in the general population–the HUNT pain study. PLoS One 2013;8:e65279. 23776464 10.1371/journal.pone.0065279 PMC3680414

[50] Lau T Horschitz S Bartsch D Schloss P Monitoring mouse serotonin transporter internalization in stem cell-derived serotonergic neurons by confocal laser scanning microscopy. Neurochem Int 2009;54:271–6. 19121357 10.1016/j.neuint.2008.12.004

[51] Lau T Schloss P Differential regulation of serotonin transporter cell surface expression. Wires Membr Transp Signal 2012;1:259–68.

[52] Lautenbacher S Rollman GB Possible deficiencies of pain modulation in fibromyalgia. Clin J Pain 1997;13:189–96. 9303250 10.1097/00002508-199709000-00003

[53] Li G Rhodes JS Girard I Gammie SC Garland T Jr Opioid-mediated pain sensitivity in mice bred for high voluntary wheel running. Physiol Behav 2004;83:515–24. 15581674 10.1016/j.physbeh.2004.09.003

[54] Lima LV DeSantana JM Rasmussen LA Sluka KA Short-duration physical activity prevents the development of activity-induced hyperalgesia through opioid and serotoninergic mechanisms. PAIN 2017 Jun 8 10.1097/j.pain.0000000000000967 [Epub ahead of print]. PMC5561491 28621702

[55] Liu YT Shao YW Yen CT Shaw FZ Acid-induced hyperalgesia and anxio-depressive comorbidity in rats. Physiol Behav 2014;131:105–10. 24726391 10.1016/j.physbeh.2014.03.030

[56] Llewelyn MB Azami J Roberts MH The effect of modification of 5-hydroxytryptamine function in nucleus raphe magnus on nociceptive threshold. Brain Res 1984;306:165–70. 6235891 10.1016/0006-8993(84)90365-2

[57] Llewelyn MB Azami J Roberts MHT Effects of 5-hydroxytryptamine applied into the nucleus raphe magnus on nociceptive thresholds and neuronal firing rate. Brain Res 1983;258:59–68. 24010164 10.1016/0006-8993(83)91226-x

[58] Manna SS Umathe SN A possible participation of transient receptor potential vanilloid type 1 channels in the antidepressant effect of fluoxetine. Eur J Pharmacol 2012;685:81–90. 22542657 10.1016/j.ejphar.2012.04.023

[59] Martins DF Mazzardo-Martins L Soldi F Stramosk J Piovezan AP Santos AR High-intensity swimming exercise reduces neuropathic pain in an animal model of complex regional pain syndrome type I: evidence for a role of the adenosinergic system. Neuroscience 2013;234:69–76. 23291454 10.1016/j.neuroscience.2012.12.042

[60] Mathes WF Kanarek RB Wheel running attenuates the antinociceptive properties of morphine and its metabolite, morphine-6-glucuronide, in rats. Physiol Behav 2001;74:245–51. 11564474 10.1016/s0031-9384(01)00577-7

[61] Mathes WF Kanarek RB Chronic running wheel activity attenuates the antinociceptive actions of morphine and morphine-6-glucouronide administration into the periaqueductal gray in rats. Pharmacol Biochem Behav 2006;83:578–84. 16712909 10.1016/j.pbb.2006.03.020

[62] Mazzardo-Martins L Martins DF Marcon R Dos Santos UD Speckhann B Gadotti VM Sigwalt AR Guglielmo LG Soares Santos AR High-intensity extended swimming exercise reduces pain-related behavior in mice: involvement of endogenous opioids and the serotonergic system. J Pain 2010;11:1393. 10.1016/j.jpain.2010.03.015 20488763

[63] Millan MJ Descending control of pain. Prog Neurobiol 2002;66:355–474. 12034378 10.1016/s0301-0082(02)00009-6

[64] Miranda A Peles S McLean PG Sengupta JN Effects of the 5-HT3 receptor antagonist, alosetron, in a rat model of somatic and visceral hyperalgesia. PAIN 2006;126:54–63. 16844296 10.1016/j.pain.2006.06.014

[65] Miranda A Peles S Rudolph C Shaker R Sengupta JN Altered visceral sensation in response to somatic pain in the rat. Gastroenterology 2004;126:1082–9. 15057747 10.1053/j.gastro.2004.01.003

[66] Nielsen AN Mathiesen C Blackburn-Munro G Pharmacological characterisation of acid-induced muscle allodynia in rats. Eur J Pharmacol 2004;487:93–103. 15033380 10.1016/j.ejphar.2004.01.017

[67] Park SH Sim YB Kang YJ Kim CH Kwon MS Suh HW The differential profiles of withdrawal symptoms induced by morphine and beta-endorphin administered intracerebroventricularly in mice. Neuroscience 2012;218:216–25. 22626645 10.1016/j.neuroscience.2012.05.010

[68] Patkar KA Wu J Ganno ML Singh HD Ross NC Rasakham K Toll L McLaughlin JP Physical presence of nor-binaltorphimine in mouse brain over 21 days after a single administration corresponds to its long-lasting antagonistic effect on kappa-opioid receptors. J Pharmacol Exp Ther 2013;346:545–54. 23853171 10.1124/jpet.113.206086 PMC13102141

[69] Paxinos G Franklin KBJ The mouse brain in stereotaxic coordinates academic press. San Diego: Academic Press, 2001.

[70] Pereira MP Donahue RR Dahl JB Werner M Taylor BK Werner MU Endogenous opioid-masked latent pain sensitization: studies from mouse to human. PLoS One 2015;10:e0134441. 26305798 10.1371/journal.pone.0134441 PMC4549112

[71] Pertovaara A Wei H Hamalainen MM Lidocaine in the rostroventromedial medulla and the periaqueductal gray attenuates allodynia in neuropathic rats. Neurosci Lett 1996;218:127–30. 8945744 10.1016/s0304-3940(96)13136-0

[72] Pierce TL Wessendorf MW Immunocytochemical mapping of endomorphin-2-immunoreactivity in rat brain. J Chem Neuroanat 2000;18:181–207. 10781736 10.1016/s0891-0618(00)00042-9

[73] Pinto M Sousa M Lima D Tavares I Participation of mu-opioid, GABA(B), and NK1 receptors of major pain control medullary areas in pathways targeting the rat spinal cord: implications for descending modulation of nociceptive transmission. J Comp Neurol 2008;510:175–87. 18615498 10.1002/cne.21793

[74] Porreca F Burgess SE Gardell LR Vanderah TW Malan TP Ossipov MH Lappi DA Lai J Inhibition of neuropathic pain by selective ablation of brainstem medullary cells expressing the mu-opioid receptor. J Neurosci 2001;21:5281–8. 11438603 10.1523/JNEUROSCI.21-14-05281.2001 PMC6762871

[75] Radhakrishnan R King EW Dickman J Richtsmeier C Schardt N Spurgin M Sluka KA Blockade of spinal 5-HT receptor subtypes prevents low, but not high, frequency TENS-induced antihyperalgesia in rats. PAIN 2003;105:205–13. 14499437 10.1016/s0304-3959(03)00207-0 PMC2746627

[76] Ramamoorthy S Giovanetti E Qian Y Blakely RD Phosphorylation and regulation of antidepressant-sensitive serotonin transporters. J Biol Chem 1998;273:2458–66. 9442097 10.1074/jbc.273.4.2458

[77] Sabharwal R Rasmussen L Sluka KA Chapleau MW Exercise prevents development of autonomic dysregulation and hyperalgesia in a mouse model of chronic muscle pain. PAIN 2016;157:387–98. 26313406 10.1097/j.pain.0000000000000330 PMC4724275

[78] Samuvel DJ Jayanthi LD Bhat NR Ramamoorthy S A role for p38 mitogen-activated protein kinase in the regulation of the serotonin transporter: evidence for distinct cellular mechanisms involved in transporter surface expression. J Neurosci 2005;25:29–41. 15634764 10.1523/JNEUROSCI.3754-04.2005 PMC6725216

[79] Sandkuhler J Gebhart GF Relative contributions of the nucleus raphe magnus and adjacent medullary reticular formation to the inhibition by stimulation of the periaqueductal gray of a spinal nociceptive reflex in the pentobarbital-anaesthetised rat. Brain Res 1984;305:77–87. 6744063 10.1016/0006-8993(84)91121-1

[80] Satoh O Omote K Roles of monoaminergic, glycinergic and GABAergic inhibitory systems in the spinal cord in rats with peripheral mononeuropathy. Brain Res 1996;728:27–36. 8864294

[81] Schul R Frenk H The role of serotonin in analgesia elicited by morphine in the periaqueductal gray matter (PAG). Brain Res 1991;553:353–7. 1681985 10.1016/0006-8993(91)90849-q

[82] Schwarz L Kindermann W Changes in beta-endorphin levels in response to aerobic and anaerobic exercise. Sports Med 1992;13:25–36. 1553453 10.2165/00007256-199213010-00003

[83] Shankarappa SA Piedras-Renteria ES Stubbs EB Jr Forced-exercise delays neuropathic pain in experimental diabetes: effects on voltage-activated calcium channels. J Neurochem 2011;118:224–36. 21554321 10.1111/j.1471-4159.2011.07302.x

[84] Sharma NK Ryals JM Liu H Liu W Wright DE Acidic saline-induced primary and secondary mechanical hyperalgesia in mice. J Pain 2009;10:1231–41. 19592308 10.1016/j.jpain.2009.04.014 PMC2787877

[85] Shmauss C Hammond DL Ochi JW Yaksh TL Pharmacological antagonism of the antinociceptive effects of serotonin in the rat spinal cord. Eur J Pharmacol 1983;90:349–57. 6688398 10.1016/0014-2999(83)90556-3

[86] Shyu BC Andersson SA Thoren P Endorphin mediated increase in pain threshold induced by long-lasting exercise in rats. Life Sci 1982;30:833–40. 7070198 10.1016/0024-3205(82)90597-5

[87] Skyba DA King EW Sluka KA Effects of NMDA and non-NMDA ionotropic glutamate receptor antagonists on the development and maintenance of hyperalgesia induced by repeated intramuscular injection of acidic saline. PAIN 2002;98:69–78. 12098618 10.1016/s0304-3959(01)00471-7

[88] Skyba DA Radhakrishnan R Rohlwing JJ Wright A Sluka KA Joint manipulation reduces hyperalgesia by activation of monoamine receptors but not opioid or GABA receptors in the spinal cord. PAIN 2003;106:159–68. 14581123 10.1016/s0304-3959(03)00320-8 PMC2732015

[89] Skyba DA Radhakrishnan R Sluka KA Characterization of a method for measuring primary hyperalgesia of deep somatic tissue. J Pain 2005;6:41–7. 15629417 10.1016/j.jpain.2004.10.002

[90] Sluka KA Berkley KJ O'Connor MJ Nicolella DP Enoka RM Boyan BD Hart DA Resnick E Kwoh CK Tosi LL Coutts RD Kohrt WM Neural and psychosocial contributions to sex differences in knee osteoarthritic pain. Biol Sex Differ 2012;3:26. 23244577 10.1186/2042-6410-3-26 PMC3583673

[91] Sluka KA Clauw DJ Neurobiology of fibromyalgia and chronic widespread pain. Neuroscience 2016;338:114–29. 27291641 10.1016/j.neuroscience.2016.06.006 PMC5083139

[92] Sluka KA Danielson J Rasmussen L Dasilva LF Exercise-induced pain requires NMDA receptor activation in the medullary raphe nuclei. Med Sci Sports Exerc 2012;44:420–7. 21795998 10.1249/MSS.0b013e31822f490e PMC3955196

[93] Sluka KA Kalra A Moore SA Unilateral intramuscular injections of acidic saline produce a bilateral, long-lasting hyperalgesia. Muscle Nerve 2001;24:37–46. 11150964 10.1002/1097-4598(200101)24:1<37::aid-mus4>3.0.co;2-8

[94] Sluka KA O'Donnell JM Danielson J Rasmussen LA Regular physical activity prevents development of chronic pain and activation of central neurons. J Appl Physiol 2013;114:725–33. 23271699 10.1152/japplphysiol.01317.2012 PMC3615604

[95] Sluka KA Price MP Wemmie JA Welsh MJ ASIC3, but not ASIC1, channels are involved in the development of chronic muscle pain. In: Dostrovsky JO Carr DB Koltzenburg M, editors. Proceedings of the 10th World Congress on Pain. Seattle: IASP Press, 2003. pp. 71–79.

[96] Sluka KA Rohlwing JJ Bussey RA Eikenberry SA Wilken JM Chronic muscle pain induced by repeated acid injection is reversed by spinally administered μ−, and δ−, but not κ−, opioid receptor agonists. J Pharmacol Exp Ther 2002;302:1146–50. 12183674 10.1124/jpet.102.033167

[97] Smith MA McClean JM Bryant PA Sensitivity to the effects of a kappa opioid in rats with free access to exercise wheels: differential effects across behavioral measures. Pharmacol Biochem Behav 2004;77:49–57. 14724041 10.1016/j.pbb.2003.09.021

[98] Smith MA Yancey DL Sensitivity to the effects of opioids in rats with free access to exercise wheels: mu-opioid tolerance and physical dependence. Psychopharmacology (Berl) 2003;168:426–34. 12709780 10.1007/s00213-003-1471-5

[99] Sorkin LS Mcadoo DJ Willis WD Release of serotonin following brainstem stimulation: correlation with inhibition of nociceptive dorsal horn neurons. In: Besson JM, editor. Serotonin and pain. Amsterdam: Elsevier, 1990 pp. 105–15.

[100] Spinella M Cooper ML Bodnar RJ Excitatory amino acid antagonists in the rostral ventromedial medulla inhibit mesencephalic morphine analgesia in rats. PAIN 1996;64:545–52. 8783320 10.1016/0304-3959(95)00192-1

[101] Stagg NJ Mata HP Ibrahim MM Henriksen EJ Porreca F Vanderah TW Philip MT Jr Regular exercise reverses sensory hypersensitivity in a rat neuropathic pain model: role of endogenous opioids. Anesthesiol 2011;114:940–8. 10.1097/ALN.0b013e318210f880 PMC6345518 21386701

[102] Tillu DV Gebhart GF Sluka KA Descending facilitatory pathways from the RVM initiate and maintain bilateral hyperalgesia after muscle insult. PAIN 2008;136:331–9. 17764841 10.1016/j.pain.2007.07.011 PMC2519171

[103] Urban MO Coutinho SV Gebhart GF Biphasic modulation of visceral nociception by neurotensin in rat rostral ventromedial medulla. J Pharmacol Exp Ther 1999;290:207–13. 10381777

[104] Urban MO Gebhart GF Characterization of biphasic modulation of spinal nociceptive transmission by neurotensin in the rat rostral ventromedial medulla. J Neurophysiol 1997;78:1550–62. 9310442 10.1152/jn.1997.78.3.1550

[105] Vanderah TW Suenaga NMH Ossipov MH Malan TP Lai J Porreca F Tonic descending facilitation from the rostral ventromedial medulla mediates opioid-induced abnormal pain and antinociceptive tolerance. J Neurosci 2001;21:279–86. 11150345 10.1523/JNEUROSCI.21-01-00279.2001 PMC6762454

[106] Vera-Portocarrero LP Xie JY Kowal J Ossipov MH King T Porreca F Descending facilitation from the rostral ventromedial medulla maintains visceral pain in rats with experimental pancreatitis. Gastroenterology 2006;130:2155–64. 16762636 10.1053/j.gastro.2006.03.025

[107] Walder RY Rasmussen LA Rainier JD Light AR Wemmie JA Sluka KA ASIC1 and ASIC3 play different roles in the development of hyperalgesia after inflammatory muscle injury. J Pain 2010;11:210–18. 20015700 10.1016/j.jpain.2009.07.004 PMC2943154

[108] Wei H Pertovaara A MK-801, an NMDA receptor antagonist, in the rostroventromedial medulla attenuates development of neuropathic symptoms in the rat. Neuroreport 1999;10:2933–7. 10549800 10.1097/00001756-199909290-00011

[109] Wiebelhaus JM Walentiny DM Beardsley PM Effects of acute and repeated administration of Oxycodone and naloxone-Precipitated withdrawal on Intracranial Self-stimulation in rats. J Pharmacol Exp Ther 2016;356:43–52. 26491062 10.1124/jpet.115.228940 PMC4702076

[110] Young EG Watkins LR Mayer DJ Comparison of the effects of ventral medullary lesions on systemic and microinjection morphine analgesia. Brain Res 1984;290:119–29. 6692127 10.1016/0006-8993(84)90741-8

[111] Zhang R Chomistek AK Dimitrakoff JD Giovannucci EL Willett WC Rosner BA Wu K Physical activity and chronic prostatitis/chronic pelvic pain syndrome. Med Sci Sports Exerc 2015;47:757–64. 25116086 10.1249/MSS.0000000000000472 PMC4324388

[112] Zhang Y Yang Z Gao X Wu G The role of 5-hydroxytryptamine1A and 5-hydroxytryptamine1B receptors in modulating spinal nociceptive transmission in normal and carrageenan-injected rats. PAIN 2001;92:201–11. 11323141 10.1016/s0304-3959(01)00259-7

[113] Zhao ZQ Chiechio S Sun YG Zhang KH Zhao CS Scott M Johnson RL Deneris ES Renner KJ Gereau RW Chen ZF Mice lacking central serotonergic neurons show enhanced inflammatory pain and an impaired analgesic response to antidepressant drugs. J Neurosci 2007;27:6045–53. 17537976 10.1523/JNEUROSCI.1623-07.2007 PMC6672267

